# Thoracoscopic repair of congenital diaphragmatic hernia in infants: a dual-suture approach using barbed and non-absorbable sutures

**DOI:** 10.3389/fped.2025.1612075

**Published:** 2025-06-18

**Authors:** Xianhui Shang, Yuanmei Liu, Zhen Luo, Yingbo Li, Guangxu Zhou, Hongyang Tan, Kaiyi Mao

**Affiliations:** ^1^ Department of Pediatric Surgery, Affiliated Hospital of Zunyi Medical University, Zunyi, China; ^2^ Department of Pediatric Surgery, Guizhou Children’s Hospital, Zunyi, China

**Keywords:** congenital diaphragmatic hernia, thoracoscopy, barbed suture, non-absorbable suture, surgical technique, pediatric surgery

## Abstract

**Objective:**

To evaluate the feasibility, safety, and clinical efficacy of a dual suture technique combining barbed suture and non-absorbable needle suture in thoracoscopic repair of congenital diaphragmatic hernia (CDH) in children.

**Methods:**

A retrospective analysis was conducted on 48 pediatric patients who underwent thoracoscopic CDH repair at our institution between March 2012 and August 2024. Based on the suturing method used, patients were divided into an observation group (barbed suture combined with non-absorbable needle suture, *n* = 21) and a control group (non-absorbable needle suture alone, *n* = 27). Perioperative indicators including operative time, intraoperative blood loss, chest tube duration, postoperative hospital stay, and arterial blood gas values (pH, PO₂, PCO₂) before and after surgery were compared between groups. Postoperative complications such as suture loosening or hernia recurrence during follow-up were also assessed.

**Results:**

All procedures were successfully completed thoracoscopically with no conversions to open surgery. Operative time was significantly shorter in the observation group compared to the control group [(37.2 ± 7.3) min vs. (65.8 ± 12.4) min]. No significant differences were found between the two groups in terms of blood loss, chest tube duration, postoperative hospital stay, or blood gas parameters (all *P* > 0.05). During a follow-up period of 6–24 months (median 12 months), no cases of suture loosening, hernia recurrence, or mortality were observed in either group.

**Conclusion:**

The dual suture technique combining barbed suture with non-absorbable needle suture is safe and effective in thoracoscopic repair of CDH in children. It significantly reduces operative time without increasing the risk of postoperative complications or recurrence. This technique is suitable for promotion in institutions with appropriate thoracoscopic expertise.

## Introduction

Congenital diaphragmatic hernia (CDH) is a developmental anomaly caused by incomplete formation of the diaphragm during fetal development, allowing abdominal organs to herniate into the thoracic cavity and resulting in abnormal thoracoabdominal anatomy (1, 2). The reported incidence of CDH ranges from 1 in 5,000–1 in 2,500 live births, with a mortality rate as high as 40%–60% (3–5).

Traditional surgical approaches include open laparotomy or thoracotomy, as well as minimally invasive techniques via laparoscopic or thoracoscopic routes. With the growing advancement of minimally invasive surgery and single-lung ventilation techniques, thoracoscopic repair has emerged as a preferred option for treating CDH due to its advantages of reduced surgical trauma, faster recovery, and improved patient acceptance (6, 7). However, the conventional thoracoscopic suturing method using non-absorbable needle sutures remains technically demanding, often requiring a longer operative time and presenting challenges in cases with large diaphragmatic defects or high-tension edges. These limitations can compromise the reliability and efficiency of the repair.

In recent years, barbed sutures have gained popularity in both laparoscopic and thoracoscopic procedures due to their unique unidirectional barbed structure, which eliminates the need for knot tying and helps evenly distribute tension across the suture line. This technique has been shown to improve both the efficiency and strength of suturing. Nevertheless, clinical evidence on the use of barbed sutures in pediatric thoracoscopic CDH repair remains limited, with few large-sample studies available to validate their effectiveness and safety.

To address this gap, the present study retrospectively analyzed 48 pediatric cases of CDH treated via thoracoscopic repair. We aimed to evaluate the clinical outcomes and safety of a dual suture technique combining barbed suture and non-absorbable needle suture, providing evidence to support its broader application in minimally invasive pediatric surgery.

## Methods

This study was approved by the Ethics Committee of the Affiliated Hospital of Zunyi Medical University, and written informed consent was obtained from the legal guardians of all enrolled patients.

We retrospectively analyzed the clinical data of 48 pediatric patients with congenital diaphragmatic hernia (CDH) who underwent thoracoscopic diaphragmatic repair at our institution between March 2012 and August 2024.

All patients were confirmed to have congenital, not traumatic or acquired, diaphragmatic hernias based on clinical history, imaging (x-ray and CT), and intraoperative findings. Cases with any history or evidence of trauma were excluded.

**Inclusion criteria** were as follows: (1) diagnosis of CDH confirmed by imaging; (2) age ≤14 years; (3) all patients underwent thoracoscopic CDH repair; and (4) complete clinical and follow-up data available.

**Exclusion criteria** included: (1) presence of severe associated congenital malformations or dysfunction of major organs; (2) preoperative infection or empyema; and (3) incomplete clinical or follow-up data. (4) diaphragmatic defects deemed to require patch repair based on intraoperative evaluation.

All included defects were considered suitable for primary repair without the need for prosthetic material. The largest defect diameter recorded intraoperatively was 3.4 cm.

Due to the retrospective nature of this study and the lack of complete standardized preoperative imaging data for all patients, we used the intraoperatively measured continuous variable of “defect diameter” to quantify defect severity. CDH classification systems (e.g., A–D types) were not uniformly available for all cases. This will be considered for inclusion in future prospective studies.

Patients were divided into two groups based on the intraoperative suturing method: the **observation group** (*n* = 21) underwent diaphragmatic repair using a dual-suture technique with barbed suture combined with non-absorbable needle suture; the **control group** (*n* = 27) received conventional interrupted suturing using non-absorbable needle sutures alone. All procedures were performed by the same experienced pediatric surgical team, adhering strictly to standard thoracoscopic surgical protocols.

**Surgical procedure:** Under general anesthesia with endotracheal intubation, patients were placed in the lateral decubitus position with the unaffected side down. One thoracoscopic port and two working ports were established on the affected side. For patients receiving two-lung ventilation, carbon dioxide (CO₂) was insufflated through the thoracoscopic port to create artificial pneumothorax. For single-lung ventilation, no CO₂ insufflation was required. Herniated abdominal organs were carefully reduced into the abdominal cavity under thoracoscopic visualization, with care taken to avoid injury to the spleen or other abdominal organs. If a hernia sac was present, it was completely reduced without resection.

**Figure 1:**
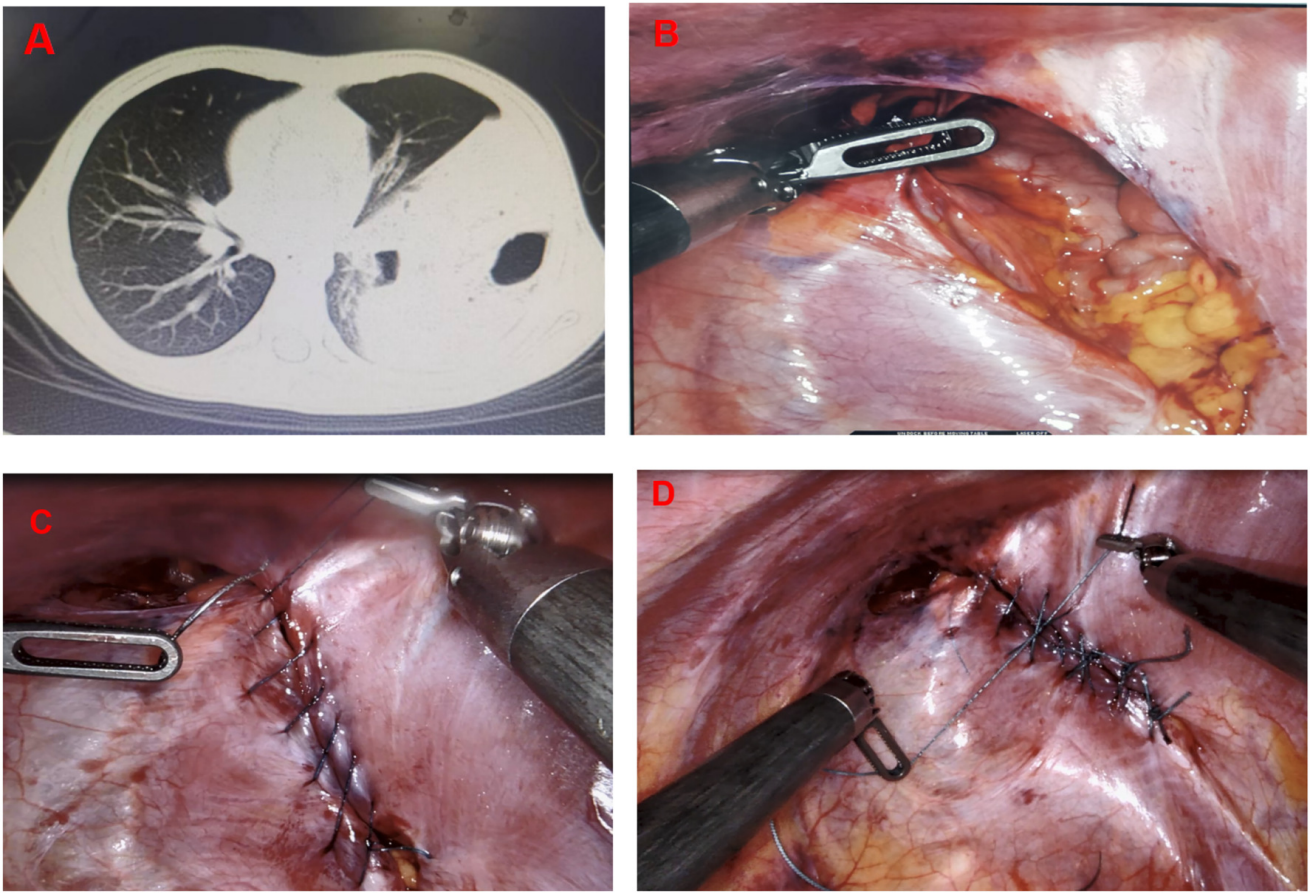
Surgical procedure of barbed suture combined with non-absorbable needle suture for thoracoscopic repair of congenital diaphragmatic hernia in children: **(A)** preoperative CT image showing herniation of abdominal contents into the thoracic cavity. **(B)** Intraoperative view revealing the diaphragmatic defect. **(C)** Continuous suturing of the defect using barbed suture to evenly reduce tension. **(D)** Reinforcement with interrupted non-absorbable needle sutures to ensure secure closure.

After delineating the edges of the diaphragmatic defect, different suturing techniques were applied according to group allocation:

**Observation group:** Continuous suturing of the defect was first performed using barbed suture to reduce tension across the diaphragmatic edge, followed by interrupted reinforcement with non-absorbable needle sutures. Suture size (4-0, 3-0, or 2-0) was selected based on patient age, diaphragmatic thickness, and defect size (Figure 1).

**Control group:** Repair was performed solely with interrupted suturing using non-absorbable needle sutures of appropriate size.

At the end of the procedure, a chest drain was routinely placed in both groups. After instrument removal, local anesthetic was injected into the intercostal spaces at the port sites, and the chest wall incisions were closed in layers.

Pulmonary hypoplasia was assessed preoperatively based on CT imaging and clinical symptoms. Diagnostic criteria included contralateral lung volume reduction, marked respiratory distress, and prolonged respiratory support requirement, in accordance with national neonatal pulmonary hypoplasia guidelines.

**Data collection:** Operative time and intraoperative blood loss were recorded during surgery. Postoperative data included chest drain duration, hospital stay, and arterial blood gas measurements [pH, partial pressure of oxygen [PO₂], and partial pressure of carbon dioxide [PCO₂]] taken before and 24 h after surgery.

All patients underwent scheduled postoperative follow-up at 1, 3, 6, 12, and 24 months. Clinical symptoms and chest imaging were reviewed to evaluate surgical outcomes and to identify any complications such as suture loosening or hernia recurrence.

**Statistical analysis** was performed using SPSS version 26.0. Continuous variables were expressed as mean ± standard deviation (x¯ ± s) and compared using independent-samples *t*-tests. Categorical variables were compared using the chi-square test. A *P*-value of <0.05 was considered statistically significant.

## Results

A total of 48 pediatric patients with congenital diaphragmatic hernia (CDH) were included in the study. Among them, 21 patients in the observation group underwent thoracoscopic repair using a dual-suture technique combining barbed suture and non-absorbable needle suture, while 27 patients in the control group received conventional interrupted suturing with non-absorbable needle sutures alone. There were no statistically significant differences between the two groups in baseline clinical characteristics such as sex, age, weight, defect size, or incidence of pulmonary hypoplasia (*P* > 0.05; Table 1).

**Table 1:** Comparison of baseline clinical characteristics between groups.

Variable	Observation group (*n* = 21)	Control group (*n* = 27)	*t*/*χ*² value	*P*-value
Sex (male/female)	12/9	15/12	0.015	0.904
Age (months)	8.7 ± 3.1	9.4 ± 3.5	0.715	0.478
Weight (kg)	6.5 ± 1.9	7.1 ± 2.2	0.989	0.328
Defect diameter (cm)	3.2 ± 0.7	3.4 ± 0.8	0.902	0.372
Pulmonary hypoplasia [*n* (%)]	3 (14.3%)	5 (18.5%)	0.151	0.698

Defect diameter was measured intraoperatively; CDH type classification (A–D) was not uniformly available due to incomplete imaging data. Pulmonary hypoplasia was defined by reduced contralateral lung volume on imaging, respiratory distress, and extended respiratory support, in line with national diagnostic criteria.

All patients successfully underwent thoracoscopic repair without conversion to open surgery. The operative time in the observation group was significantly shorter than that in the control group (37.2 ± 7.3 min vs. 65.8 ± 12.4 min, *P* < 0.05), indicating a statistically significant difference. No significant differences were observed between the two groups in terms of intraoperative blood loss, chest tube duration, or postoperative hospital stay (*P* > 0.05; Table 2).

**Table 2:** Comparison of perioperative indicators between groups.

Indicator	Observation group (*n* = 21)	Control group (*n* = 27)	*t*-value	*P*-value
Operative time (min)	37.2 ± 7.3	65.8 ± 12.4	13.521	<0.001
Intraoperative blood loss (ml)	4.3 ± 1.2	4.8 ± 1.5	1.256	0.215
Chest tube duration (days)	3.1 ± 0.7	3.4 ± 0.8	1.366	0.179
Postoperative hospital stay (d)	6.5 ± 1.2	6.9 ± 1.4	1.051	0.299

Arterial blood gas parameters, including pH, PO₂, and PCO₂, measured preoperatively and at 24 h postoperatively, showed no significant differences between the two groups (*P* > 0.05; Table 3).

**Table 3:** Comparison of arterial blood gas parameters before and after surgery.

Parameter	Time point	Observation group (*n* = 21)	Control group (*n* = 27)	*t*-value	*P*-value
pH	Pre-op	7.38 ± 0.04	7.37 ± 0.05	0.757	0.453
	24 h Post-op	7.36 ± 0.05	7.35 ± 0.06	0.626	0.534
PO₂ (mmHg)	Pre-op	85.3 ± 6.4	83.7 ± 7.2	0.803	0.426
	24 h Post-op	91.5 ± 7.1	89.8 ± 8.3	0.749	0.457
PCO₂ (mmHg)	Pre-op	39.2 ± 4.1	40.5 ± 4.8	0.997	0.324
	24 h Post-op	38.6 ± 3.7	39.8 ± 4.2	1.036	0.306

**Table 4:** Comparative advantages of the dual-suture technique vs. traditional method.

Parameter	Barbed + Non-absorbable Suture	Non-absorbable Suture Only
Operative time	Significantly shorter	Relatively longer
Intraoperative blood loss	Minimal	Minimal
Technical difficulty	Relatively low, short learning curve	High, steep learning curve
Suture security	High, evenly distributed tension	Operator-dependent
Risk of complications/recurrence	Very low	Low
Recommended clinical application	Highly recommended	Optional

All patients were followed for 6–24 months, with a median follow-up of 12 months. During the follow-up period, no cases of suture loosening, hernia recurrence, or other serious complications were observed in either group. No mortality occurred.

## Conclusion

The findings of this study demonstrate that the dual-suture technique combining barbed suture with non-absorbable needle suture is a safe and effective approach for thoracoscopic repair of congenital diaphragmatic hernia in children. Compared to the conventional technique using non-absorbable needle sutures alone, this method significantly shortens operative time without increasing intraoperative blood loss, postoperative recovery time, or complication rates. Moreover, no cases of recurrence or suture failure were observed during short- to mid-term follow-up.

Given its procedural simplicity and ease of adoption, especially for surgeons with basic thoracoscopic experience, this technique offers a valuable optimization for minimally invasive CDH repair and holds strong potential for wider clinical application (Table 4).

## Discussion

Congenital diaphragmatic hernia (CDH) typically develops before 10 weeks of gestation. As the fetus continues to grow, the herniation of abdominal organs into the thoracic cavity increases intrathoracic pressure, impairs normal lung development, and disrupts fetal respiratory movements. Pulmonary hypoplasia associated with CDH is considered a developmental defect that originates during the embryonic stage. Traditionally, early postnatal surgical intervention has been advocated to relieve the compression of mediastinal structures and lungs by the herniated organs, restore normal anatomy, and improve cardiopulmonary function in affected infants (8–12).

Thoracoscopic repair of CDH offers several advantages, including minimally invasive access, enhanced visualization of the operative field, reduced surgical trauma, shorter hospital stay, and lower overall medical costs, thereby promoting faster recovery (13–16). Some studies have also suggested that thoracoscopic surgery enables better visualization of the diaphragmatic defect, shortens operative time, and facilitates rapid postoperative recovery, making it a preferred approach for neonatal CDH repair (17–19). While various surgical techniques have been developed for CDH, comparative studies evaluating their outcomes remain important.

In this study, we demonstrated that the dual-suture technique combining barbed suture with non-absorbable needle suture significantly reduced operative time in pediatric thoracoscopic CDH repair, without increasing intraoperative blood loss, chest tube duration, or length of hospital stay. Compared to the conventional method using non-absorbable needle sutures alone, the dual-suture approach provided notable perioperative advantages.

The conventional thoracoscopic technique for CDH repair typically employs non-absorbable needle sutures alone. Although this method has been used extensively in clinical practice, it is technically demanding and associated with greater suture tension. In cases of large defects or thin diaphragmatic tissue, this approach may carry a higher risk of intraoperative knot loosening or postoperative recurrence. Barbed sutures, as a novel material, feature unidirectional barbs that anchor the suture in tissue, effectively distributing tension along the suture line and eliminating the need for knot tying. In our study, barbed sutures were used for initial continuous closure to reduce tension, followed by interrupted reinforcement with non-absorbable needle sutures, yielding more secure and efficient suture fixation.

Recent studies have increasingly highlighted the utility of barbed sutures in pediatric minimally invasive surgery. Lukish et al. first demonstrated their feasibility in children undergoing laparoscopic procedures, noting reduced operative complexity and knotless closure (20). More recently, Muensterer et al. reported the clinical application of barbed sutures specifically in CDH repair, reinforcing their potential for secure and efficient diaphragmatic closure (21). Furthermore, Shehata et al. conducted a comparative study showing favorable outcomes for barbed sutures vs. conventional interrupted techniques in thoracoscopic CDH repair (22). These findings align with our experience and further support the safety and practicality of barbed suture application in this context. Our dual-suture strategy builds upon this foundation, providing additional reinforcement and minimizing the risk of suture failure during the learning curve or in higher-tension defects.

Although barbed sutures offer clear advantages such as knotless anchoring and uniform tension distribution, they were not used as a standalone technique in this study. At the time of study initiation, there was insufficient long-term evidence to confirm the durability of barbed sutures alone in pediatric CDH repair. As a result, we adopted a hybrid approach—barbed sutures for initial closure followed by reinforcement with non-absorbable sutures—to prioritize surgical safety and suture reliability. This conservative strategy also ensured reproducibility across different surgeons during the learning phase of adopting barbed suture technology. 333333 We acknowledge that in small, low-tension defects, barbed sutures alone may potentially be sufficient. Future studies with extended follow-up and larger sample sizes may further assess the feasibility of barbed sutures as a standalone modality in pediatric thoracoscopic CDH repair.

The rationale for adopting this dual-suture approach was threefold. First, from a safety perspective, reinforcement with non-absorbable sutures provides additional stability, even in small defects, helping to prevent postoperative dehiscence. Second, considering the learning curve associated with barbed suture application in pediatric thoracoscopy, this hybrid technique offered a safer transition for surgeons acquiring experience with the new material. Third, we observed that in certain cases classified as morphologically small (potentially type A defects), the diaphragmatic tissue was thin or subjected to uneven tension; reinforcement allowed for improved load distribution and suture reliability under these circumstances. Although barbed sutures alone may suffice in select low-tension repairs, our protocol prioritized reproducibility and consistency across the patient cohort. This decision may have contributed to the complete absence of suture loosening or recurrence during follow-up.

Given the limited intrathoracic working space and technical difficulty of pediatric thoracoscopic procedures, evaluating the practical application of barbed sutures in this population is clinically meaningful. In our observation group, no cases of knot loosening or recurrence were observed during follow-up, further supporting the reliability of this technique in pediatric CDH repair.

Although the inclusion criterion allowed for patients up to 14 years of age, the vast majority of our cohort were infants, with mean ages of 8.7 ± 3.1 months and 9.4 ± 3.5 months in the two groups. A small number of older patients were included due to delayed diagnosis of congenital diaphragmatic hernia. These cases were not acquired or traumatic hernias; rather, they were confirmed congenital defects that had not been previously identified or treated. All such cases were thoroughly evaluated to exclude any history of trauma, and imaging and intraoperative assessments were consistent with congenital etiology.

One limitation of our study is the lack of standardized defect classification using systems such as the CDH Study Group's A–D typing. Due to the retrospective design and incomplete availability of preoperative imaging in some cases, we used intraoperatively measured “defect diameter” as a continuous variable to quantify defect severity. While this approach provides objective and direct data, it may limit comparability with studies that use categorical defect classification. Future prospective studies should aim to incorporate both quantitative measurements and standardized classification systems to enhance data consistency and cross-study comparability.

None of the patients in our cohort required patch repair, which reflects the relatively small defect sizes observed at our center. This may limit the generalizability of our findings to patients with larger or more complex defects. Future studies should include broader case selection to evaluate whether the dual-suture technique remains effective in defects requiring patch reinforcement.

Nevertheless, this study has certain limitations. First, it is a single-center retrospective study with a relatively small sample size, which may introduce selection bias. Second, the maximum follow-up period was 24 months, and long-term outcomes beyond this period remain unclear. Future multicenter prospective randomized controlled studies with larger sample sizes are needed to validate our findings.

## Conclusion

The dual-suture technique combining barbed suture and non-absorbable needle suture appears to be a safe and effective method for thoracoscopic repair of congenital diaphragmatic hernia (CDH) in infants with small to moderate defect sizes. In our cohort, this approach significantly reduced operative time and technical complexity, without increasing the risk of postoperative complications or recurrence. While the findings are encouraging, they are based on a population predominantly composed of delayed-diagnosed, isolated CDH cases without severe comorbidities. Further prospective studies involving neonatal cases and a broader spectrum of defect types are warranted to validate the generalizability of this technique. Nonetheless, given its technical feasibility, this dual-suture strategy holds promise for broader clinical application in pediatric centers with thoracoscopic expertise.

## Data Availability

The original contributions presented in the study are included in the article/Supplementary Material, further inquiries can be directed to the corresponding author.
